# Indoxyl Sulfate-Mediated Metabolic Alteration of Transcriptome Signatures in Monocytes of Patients with End-Stage Renal Disease (ESRD)

**DOI:** 10.3390/toxins12100621

**Published:** 2020-09-28

**Authors:** Hee Young Kim, Su Jeong Lee, Yuri Hwang, Ga Hye Lee, Chae Eun Yoon, Hyeon Chang Kim, Tae-Hyun Yoo, Won-Woo Lee

**Affiliations:** 1 Department of Microbiology and Immunology, Seoul National University College of Medicine, Seoul 03080, Korea; hyk0801@hotmail.com (H.Y.K.); yuri.hwang@genexine.com (Y.H.); zcbtcey@ucl.ac.uk (C.E.Y.); 2 Institute of Infectious Diseases, Seoul National University College of Medicine, Seoul 03080, Korea; 3 Laboratory of Inflammation and Autoimmunity (LAI), Department of Biomedical Sciences and BK21Plus Biomedical Science Project, Seoul National University College of Medicine, Seoul 03080, Korea; minyahile@naver.com (S.J.L.); icefallv@hanmail.net (G.H.L.); 4 Cardiovascular and Metabolic Diseases Etiology Research Center and Department of Preventive Medicine, Yonsei University College of Medicine, Seoul 03722, Korea; HCKIM@yuhs.ac; 5 Division of Nephrology, Department of Internal Medicine, Yonsei University College of Medicine, Seoul 03722, Korea; YOOSY0316@yuhs.ac; 6 Cancer Research Institute and Ischemic/Hypoxic Disease Institute, Seoul National University College of Medicine, Seoul National University Hospital Biomedical Research Institute, Seoul 03080, Korea

**Keywords:** end-stage renal disease (ESRD), monocytes, uremic toxins, indoxyl sulfate (IS), aryl hydrocarbon receptor, transcriptomic analysis, metabolic pathway

## Abstract

End-stage renal disease (ESRD) is the final stage of chronic kidney disease, which is increasingly prevalent worldwide and is associated with the progression of cardiovascular disease (CVD). Indoxyl sulfate (IS), a major uremic toxin, plays a key role in the pathology of CVD via adverse effects in endothelial and immune cells. Thus, there is a need for a transcriptomic overview of IS responsive genes in immune cells of ESRD patients. Here, we investigated IS-mediated alterations in gene expression in monocytes from ESRD patients. Transcriptomic analysis of ESRD patient-derived monocytes and IS-stimulated monocytes from healthy controls was performed, followed by analysis of differentially expressed genes (DEGs) and gene ontology (GO). We found that 148 upregulated and 139 downregulated genes were shared between ESRD patient-derived and IS-stimulated monocytes. Interaction network analysis using STRING and ClueGo suggests that mainly metabolic pathways, such as the pentose phosphate pathway, are modified by IS in ESRD patient-derived monocytes. These findings were confirmed in IS-stimulated monocytes by the increased mRNA expression of genes including G6PD, PGD, and TALDO1. Our data suggest that IS causes alteration of metabolic pathways in monocytes of ESRD patients and, thus, these altered genes may be therapeutic targets.

## 1. Introduction

Chronic kidney disease (CKD) is a prevalent disease of increasing frequency worldwide. Based on the estimated glomerular filtration rate (eGFR), CKD is categorized into five stages [1] and patients diagnosed with the final stage, stage 5 (eGFR < 15 mL/min) also known as end-stage renal disease (ESRD), require hemodialysis or kidney transplantation. Despite the advance of current therapeutic technologies, the mortality rate of ESRD patients has reached 20% [2]. The main causes of morbidity and mortality of patients with ESRD are cardiovascular diseases (CVD) and infections, with mortality rates of 50% and 20%, respectively [3,4]. Classical risk factors of CVD including diabetes, dyslipidemia and high blood pressure, cannot entirely account for the increased cardiovascular risk in ESRD patients [5,6]. In addition, the control of traditional risk factors, such as obesity, hypertension, and cholesterol as well as treatments such as statins for CVD, only show partial protection from cardiovascular events [5,7,8,9]. Based on this fact, it is assumed that other factors are important in the progression of CVDs in ESRD patients.

Progressive renal failure in CKD causes the accumulation of uremic toxins, which is intimately associated with high cardiovascular risk and mortality due to oxidative stress and augmented cytokine milieu caused by these molecules [10,11]. Among approximately 100 uremic toxins identified so far in patients with CKD [10], indoxyl sulfate (IS) and *p*-cresyl sulfate (PCS), tryptophan and tyrosine derivatives, respectively, are major uremic toxins derived from gut microbiota and are metabolized via microbial fermentation. In ESRD patients, the elevated risk of CVD is closely related to uremia-related immune activation, for instance, hypercytokinemia and inflammation [12,13,14]. IS and PCS are also well-known inducing factors of inflammatory cytokine and chemokine production in vascular endothelial cells (VECs) and vascular smooth muscle cells (VSMCs). A hallmark of CKD-related CVD is intravascular macrophage and T-cell mediated inflammation [15]; however, uremic toxin-mediated immune dysfunctions are poorly understood. Our previous study suggested that IS-induced TNF-α production by monocytes in ESRD patients causes endothelial damage through the recruitment and expansion of the cytotoxic CD4^+^CD28^−^ T cell population [16]. Moreover, IS-induced TNF-α production in human macrophages is intricately regulated by crosstalk between the aryl hydrocarbon receptor (AhR), NF-κB, and SOCS2. Likewise, IS-activated macrophages also contribute to the progression of atherosclerosis by induction of proinflammatory cytokines via Notch signaling [17].

Transcriptome analysis enables the identification of potential pathogenic drivers of disease and biological targets for treatment [18]. Despite accumulating evidence that monocytes/macrophages play a pivotal role in the pathogenesis of CVD in ESRD patients, current knowledge of transcriptomic signatures of monocytes or macrophages in ESRD patients is lacking, and data, if any, comes mainly from analysis of the average gene expression in bulk peripheral blood mononuclear cells (PBMC)s [19,20,21,22]. Moreover, little is known about how much the major uremic toxins, IS and PCS, affect gene expression in monocytes of ESRD and CKD patients.

In the present study, we sought to unveil alterations in gene expression and biological pathways mediated by major uremic toxins, such as IS and PCS, in ESRD patient-derived monocytes via microarray and interaction network analysis.

## 2. Results

### 2.1. Transcriptomic Analysis of Monocytes Derived from ESRD Patients and Monocytes Stimulated with Major Uremic Toxins, IS and PCS

**Figure 1:**
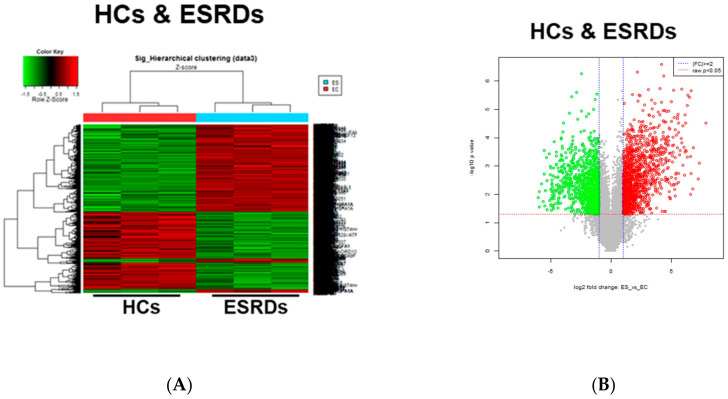

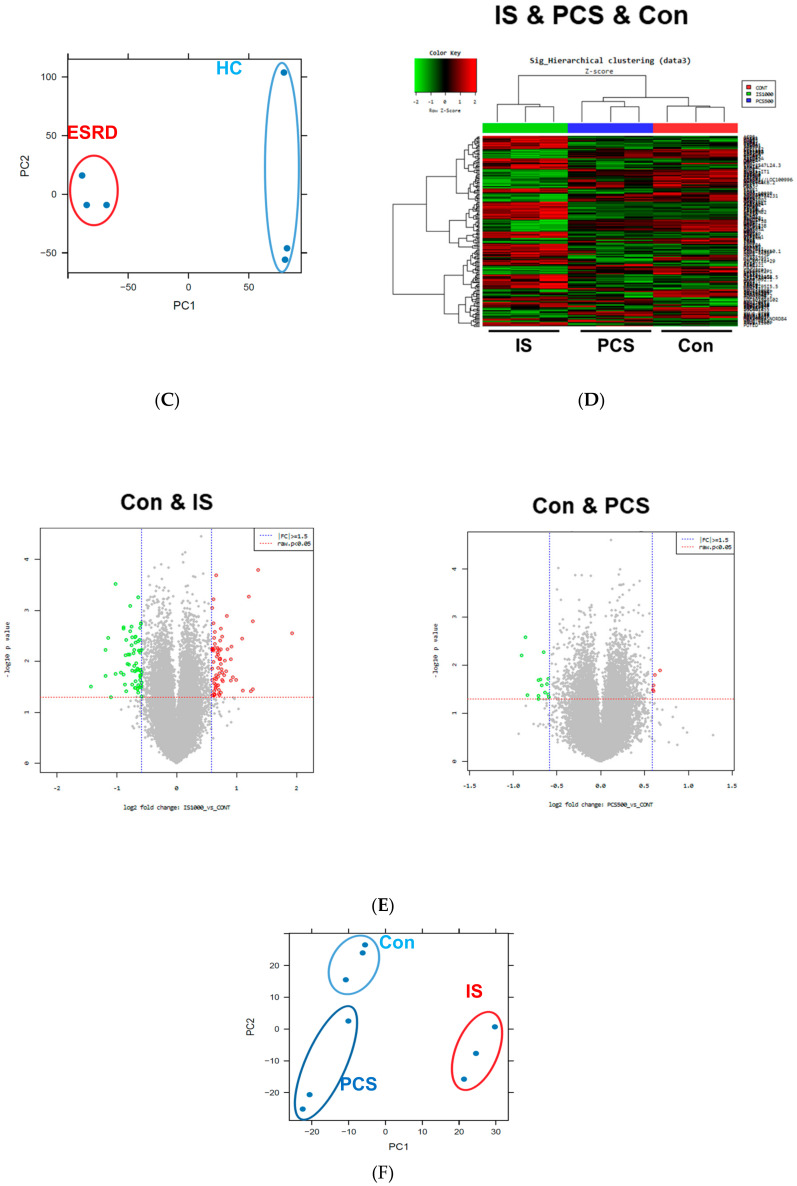
Transcriptomic profiles in End-Stage Renal Disease (ESRD) patient-derived monocytes and indoxyl sulfate (IS)- or *p-*cresyl sulfate (PCS)-stimulated monocytes derived from healthy controls (HCs). (**A**–**C**), The CD14^+^ monocytes were separated from ESRD and HC patient peripheral blood mononuclear cells (PBMCs) using magnetic beads (*n* = 3). Total RNA was purified and analyzed by microarray. Heatmap (**A**), volcano plot (**B**), and principle component analysis (PCA) (**C**) are presented. (**D**,**E**), Monocytes separated from HCs were treated with IS 1000 μM or PCS 500 μM for 24 h (*n* = 3), and then purified total RNA was subjected to microarray analysis. Heatmap of differentially expressed genes (DEGs) (**D**), volcano plot (**E**), and PCA (**F**) were analyzed among IS-treated, PCS-treated, and control (Con) monocytes.

CKD is associated with significant increases in CVDs including atherosclerosis, the development of which occurs as a result of the local inflammatory milieu and resulting effects on the arterial wall [6]. Further, retained uremic toxins are believed to be an underlying cause of the proinflammatory cytokine milieu and concomitantly impaired immune system seen in CKD. Our previous study demonstrated that in ESRD patients, monocytes respond to IS through the AhR and consequently produce increased levels of TNF-α. Given the critical role of monocytes in the pathogenesis of CKD, it is important to understand uremic toxin-mediated changes in gene expression in monocytes. To investigate the alteration of gene expression in monocytes derived from ESRD patients, we performed microarray analysis on freshly purified monocytes from peripheral blood of three ESRD patients and three healthy controls (HCs). The demographic characteristics of ESRD patients are presented in Supplementary Table S1. As a result, we identified differentially expressed genes (DEGs) including 3164 upregulated genes and 3466 downregulated genes in ESRD patient-derived monocytes compared to HCs (*p* < 0.05, FDR < 0.25). Further analysis to compare the transcriptomic profiles of the two groups using heatmaps, volcano plots, and principle component analysis (PCA) (Figure 1A–C) clearly illustrate the substantial amount of transcriptomic alterations in ESRD patient-derived monocytes compared to those of HCs. Thus, this suggests that these changes are potentially attributable to the chronic exposure to uremic toxins. IS and PCS, which originate from dietary amino acid metabolites of colonic microbial organisms, are the two most problematic uremic toxins, conferring renal and cardiovascular toxicity by increased inflammation, calcification, and oxidative stress [11,23,24,25]. To explore uremic toxin-related changes in gene expression in ESRD patient-derived monocytes, purified monocytes from healthy controls were treated with IS or PCS for 24 h followed by microarray analysis of these samples. DEGs in IS-stimulated monocytes consist of 713 upregulated and 886 downregulated genes compared to control monocytes (Figure 1D–F). Of interest, a limited range of transcriptomic changes was observed in PCS-stimulated monocytes (138 upregulated and 132 downregulated genes) as shown in the heatmap, volcano plot, and PCA (Figure 1D–F), implying that altered gene expression in ESRD patient-derived monocytes is associated more with the effects of IS than PCS. Furthermore, these results suggest that the uremic milieu is related to the alteration of the transcriptomic profile in ESRD patient-derived monocytes.

### 2.2. Common Differentially Expressed Genes (DEGs) between ESRD Patient-Derived Monocytes and IS-Stimulated Monocytes

**Figure 2:**
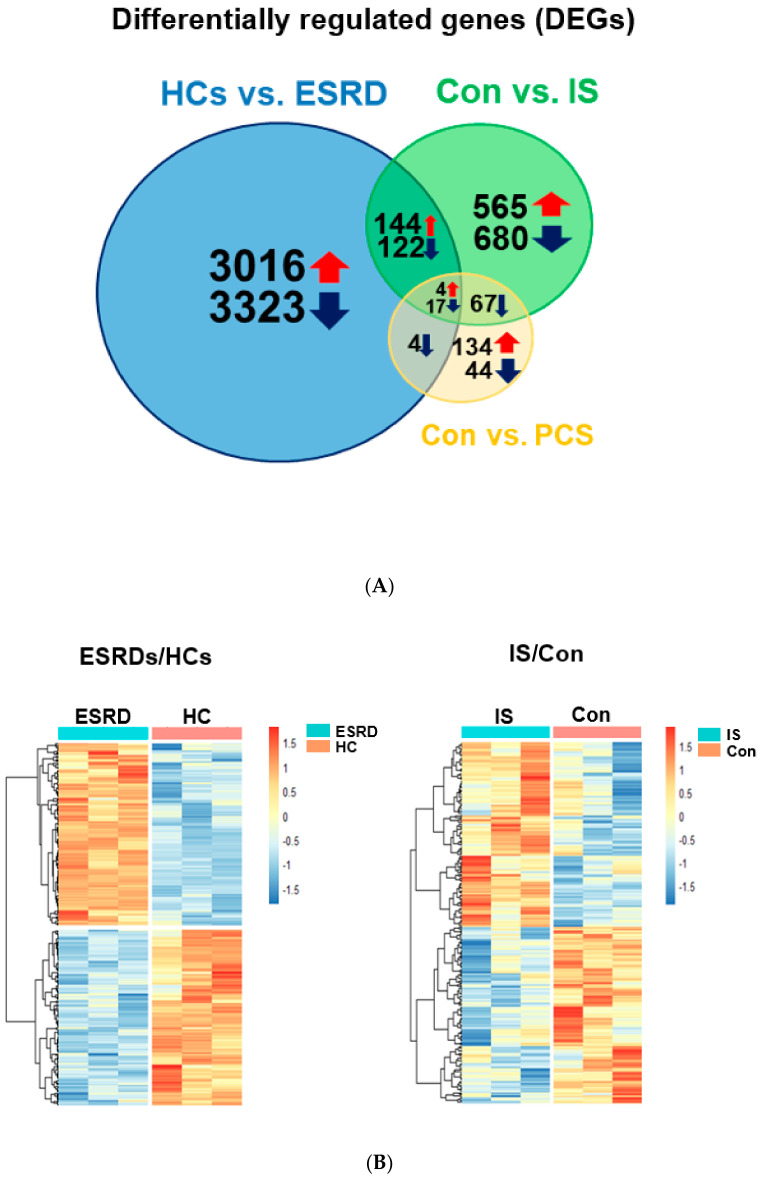
Differentially expressed genes (DEGs) common between ESRD patient-derived monocytes and IS- or PCS-stimulated monocytes derived from HCs. (**A**), Comparative analysis using Venn diagrams to identify the number of shared DEGs among those (*p* < 0.05, FDR < 0.25) obtained from each microarray analysis. (**B**), Heatmap of 148 upregulated and 139 downregulated shared DEGs between ESRD patient-derived monocytes and IS-stimulated monocytes shown in (**A**).

To further elucidate IS-mediated alteration of gene expression in ESRD patient-derived monocytes, we conducted a comparative analysis using Venn diagrams which allows for identification of the number of shared differentially expressed genes (DEGs) among ESRD-patient derived monocytes, IS- or PCS-treated monocytes (Figure 2A). This revealed that 148 upregulated and 139 downregulated genes were shared DEGs between ESRD patient-derived monocytes and IS-treated monocytes (Figure 2). Only 25 genes out of 270 DEGs in monocytes treated with PCS were shared with DEGs in ESRD patient-derived monocytes (Figure 2A), suggesting a minimal effect of PCS on regulating transcriptional levels in monocytes of ESRD patients. Our preliminary study also showed no notable impact on production of proinflammatory cytokines such as TNF-α and IL-1β in PCS-treated monocytes compared with IS-treated monocytes, although both uremic toxins accumulate significantly in the plasma of ESRD patients [16]. Thus, we decided to focus our analysis on the effects of IS on the alteration of gene expression in monocytes and its putative roles in ESRD patient-derived monocytes. Expression profiling of a total of 287 shared genes between DEGs in monocytes derived from ESRD patients and IS-treated monocytes was visualized as heatmaps (Figure 2B) and major shared genes were listed according to the log fold change in monocytes of patients with ESRD compared to HCs (Table 1 and Table S2). As reported in our previous study, representatively upregulated genes in the top 50 (Table 1) included CYP1B1, a typical AhR-responsive and xenobiotic metabolism-related gene, and TNF-α, a major proinflammatory cytokine produced by IS-treated monocytes. This data suggests that our microarray analysis fairly reflects the changes in gene expression in ESRD patients in the uremic toxin-rich milieu. Our results show that the number of DEGs shared with ESRD patient-derived monocytes is higher in IS-stimulated monocytes than in those stimulated with PCS. Thus, this implies that IS is a major uremic toxin involved in alteration of gene expression in ESRD patient-derived monocytes.

### 2.3. Principle Enriched Pathways in ESRD Patient-Derived Monocytes

**Figure 3:**
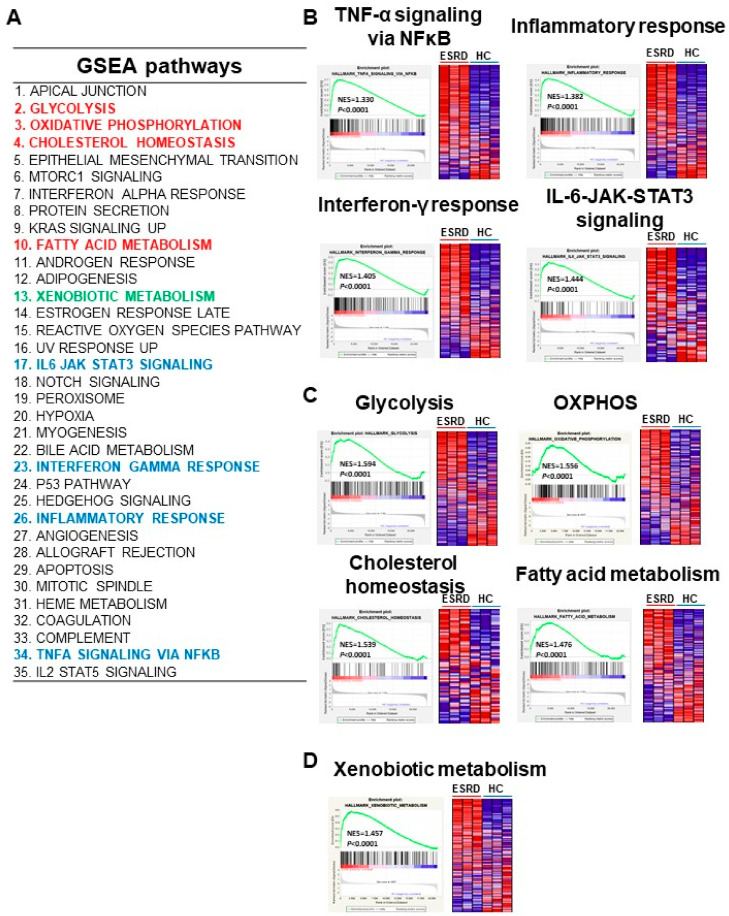
Gene set enrichment analysis (GSEA) of DEGs in ESRD patient-derived monocytes. (**A**), The 35 enriched pathways obtained from 6630 DEGs in monocytes of ESRD patients are listed (*p* < 0.05, FDR < 0.25). (**B**–**D**), GESA plots of selected immune response-related pathways (**B**), metabolic pathways (**C**), and xenobiotic metabolism pathway (**D**) are shown in monocytes from ESRD patients vs. healthy controls. Left panels show GSEA enrichment plots (score curves) and the heatmap on the right side of each panel is a visualization of the genes contributing the most to the enriched pathway or biological process.

To gain mechanistic insight into the genes found in our microarray analysis by identifying the principle enriched pathways, gene set enrichment analysis (GSEA) was performed on DEGs in ESRD patient-derived monocytes using the Gene Set Enrichment Analysis (GSEA) Molecular Signatures Database (MsigDB). This analysis is a computational method that detects modest, but coordinated, changes in the expression of groups of functionally related genes [26]. We first ranked all genes according to the extent of their differential expression in monocytes derived from ESRD patients and healthy controls. We then computed normalized enrichment scores (NES) for a collection of 50 curated hallmark gene sets representing canonical biological pathways, and identified gene sets overrepresented at both extremes of the ranked list. As presented in Figure 3A, there were 35 significantly enriched pathways obtained from 6630 DEGs in monocytes derived from ESRD patients (*p* < 0.05 and FDR < 0.25) (Figure 3A and Table S3). Immune response-related GSEA pathways such as IFN-γ response, IL-6/JAK/STAT3 signaling, inflammatory response, and TNF-α signaling via NF-κB were included (Figure 3A,B). Of note, the top 10 enriched pathways incorporated major metabolic pathways such as glycolysis, oxidative phosphorylation, cholesterol homeostasis, fatty acid metabolism, and mTORC1 signaling, suggesting that metabolic alterations affect immune functions in monocytes of ESRD patients (Figure 3A,C and Figure S1A). Moreover, a pathway related to xenobiotic metabolism, specifically involved in removal of xenobiotics via cytochrome p450, was enriched in ESRD patient-derived monocytes (Figure 3A,D). We generated GSEA enrichment plots and heatmaps of DEGs belonging to metabolic pathways and inflammatory pathways enriched in monocytes derived from ESRD patients (Figure 3B–D and Figure S1). We also identified the IS-induced notch signaling pathway, which was recently reported to mediate vascular inflammation in CKD via Delta-like 4 (DII4) [17], as an enriched pathway (Figure 3A and Figure S1B). Thus, this analysis revealed that metabolic pathways, including glycolysis and oxidative phosphorylation, were the main pathways affected in ESRD patient-derived monocytes exposed to uremic milieu.

### 2.4. Interaction Network Analysis of Common DEGs comparing ESRD Patient-Derived Monocytes and IS-Treated Monocytes

**Figure 4:**
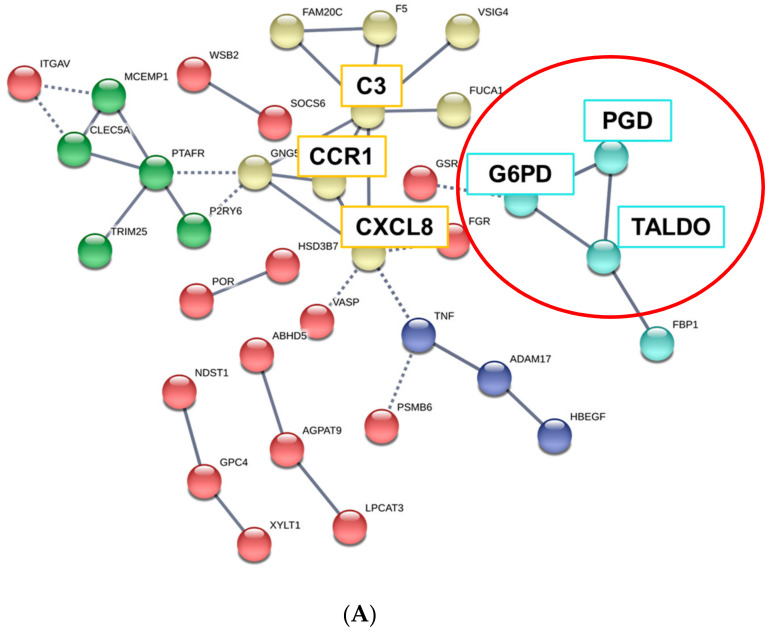

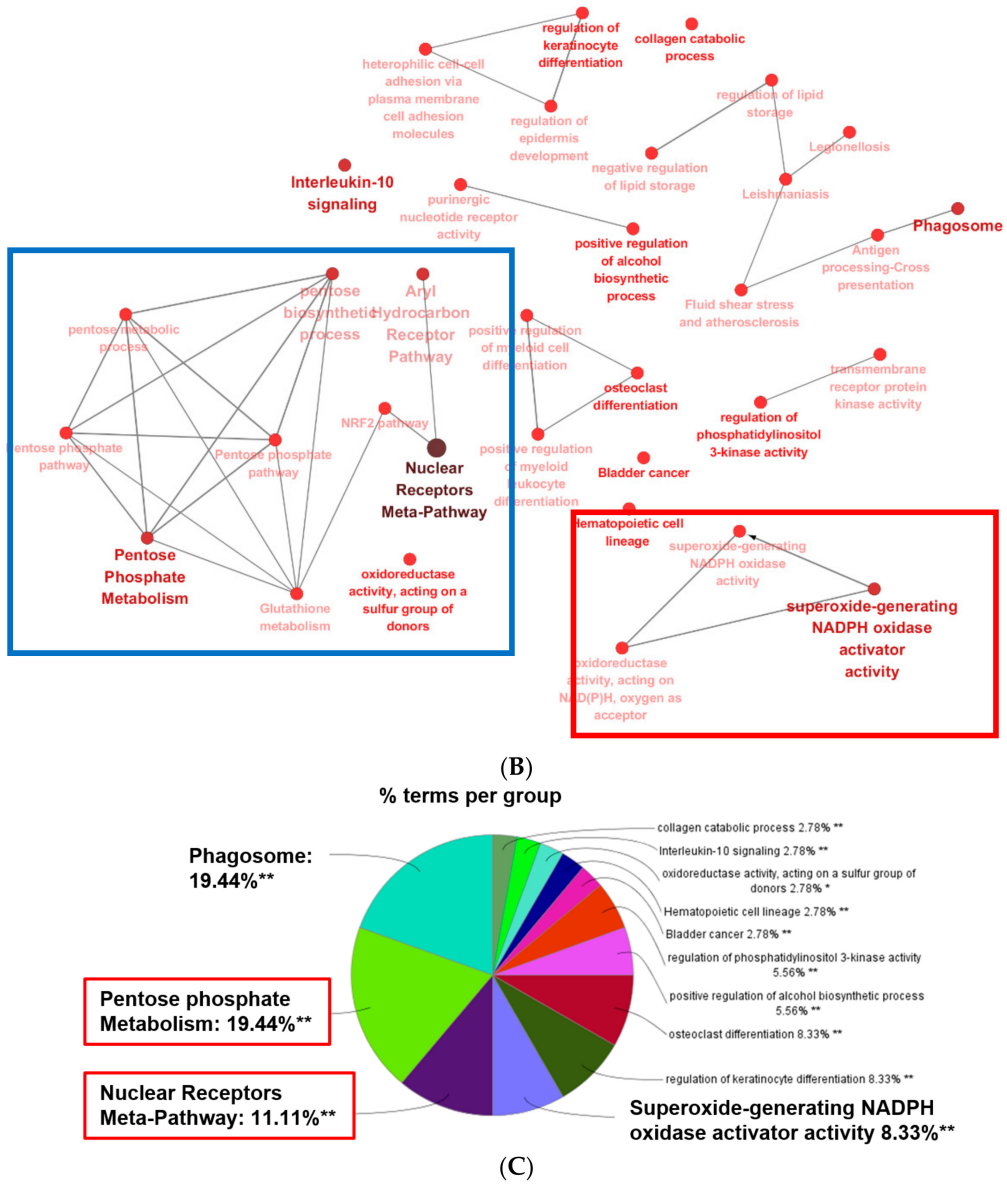
GO analysis of protein-protein interaction (PPI) networks for commonly upregulated DEGs in ESRD patient-derived monocytes and ex vivo IS-stimulated monocytes. (**A**), STRING analysis (Ver. 11.0) showing the predicted protein–protein interaction (PPI) networks among 148 selected upregulated monocyte genes. The average node degree was 0.544 and the local clustering coefficient average was 0.191. The network nodes represent each protein, while edges represent pair-wise protein interactions, and line thickness indicates the strength of data support. Each node color represents a specific biomolecular pathway. Red circles indicated genes related to PPP. (**B**), ClueGO analysis of IS-related biological pathways in monocytes of ESRD patients. Functionally grouped network of enriched categories was visualized by Cytoscape Plugin using 148 upregulated DEGs in Figure 2. Each node is a gene ontology (GO) biomolecular pathway and the edges show connectivity between nodes with regard to the functional linkage of biological processes. The node size represents the term enrichment significance. Color code represents the statistical significance, where darker colors indicate higher significance of the GO group. Blue rectangles indicate the pentose phosphate metabolism and AhR pathways, and red rectangles represent NADPH oxidase activity. (**C**), The node pie chart represents the molecular function of shared upregulated DEGs. Enrichment score (%) indicates the upregulated DEGs involved in enriched Go biological processes. * *p* < 0.05, **, *p* < 0.01 indicates the significant terms in the group.

We next constructed the interaction network between common upregulated or downregulated DEGs in ESRD patient-derived monocytes and IS-treated monocytes via STRING and ClueGo analysis. The physical or functional protein-protein interaction (PPI) network was constructed and visualized using web-based STRING (Protein-Protein Interaction Networks Functional Enrichment Analysis) software (Figure 4A and Figure S2A). In the PPI network of 148 commonly upregulated DEGs, there were 35 protein nodes, which formed five distinct clusters with 28 edges under the application of a highest confidence (0.900). The average node degree was 0.544, and the local clustering coefficient average was 0.191 (Figure 4A, unconnected nodes hidden); whereas 139 common DEGs downregulated in ESRD patient-derived monocytes and IS-treated monocytes were separated into three distinct networks with 17 protein nodes (Figure S2A, unconnected nodes hidden). The networks analyzed with 148 commonly upregulated DEGs consisted of 150 significantly enriched gene ontology (GO) biological processes (FDR < 0.05). Of these, GO terms describing response to stimuli and metabolic processes related to 86 and 92 genes, respectively (data not shown). TNF-α, CXCL8, CCR1, and C3 were closely interconnected and each of their networks was further expanded by additional proteins (Figure 4A). Of interest, a STRING-generated interaction network revealed three genes, G6PD, PGD and TALDO1, involved in pentose biosynthetic processes were significantly enriched and strongly associated with each other in monocytes derived from ESRD patients and IS-treated monocytes (Figure 4A, red circle). In contrast, analysis of commonly downregulated DEGs revealed 15 significantly enriched GO biological process (FDR < 0.05), including chromatin silencing and chromatin organization-associated gene sets with 9 and 12 genes, respectively (Figure S2A and data not shown), implying that the expression of many metabolic and inflammatory genes may be increased. To further decipher functionally grouped gene ontology and pathway annotation networks, we also conducted ClueGo analysis allowing the visualization of terms corresponding to a list of genes and for comparing functional annotations of two clusters (Figure 4B). As observed in GSEA and STRING analysis (Figure 3 and Figure 4A), the pentose phosphate pathway (PPP) and AhR-associated pathway were identified with ClueGo analysis (Figure 4B, blue rectangle). In addition, the superoxide generating nicotinamide adenine dinucleotide phosphate (NADPH) oxidase activator activity was also identified as a major pathway (Figure 4B, red rectangle). To evaluate our results, enriched GO terms were investigated. Enrichment scores were higher in phagosome (19.44%), PPP (19.44%), nuclear receptor meta-pathway (11.11%), and NADPH oxidase activator activity (8.33%) (Figure 4C), as seen in our previous analysis. Further, ClueGo analysis of 139 downregulated DEGs revealed the importance of histone modification-related pathways (94.44%; Figure S2B,C). Taken together, STRING and ClueGO analysis illustrates that IS is a major uremic toxin responsible for inducing a variety of genes related to metabolic pathways, the AhR nuclear receptor pathway, and inflammatory pathways in ESRD patient-derived monocytes.

### 2.5. Experimental Validation of DEGs in IS-Treated Monocytes

**Figure 5:**
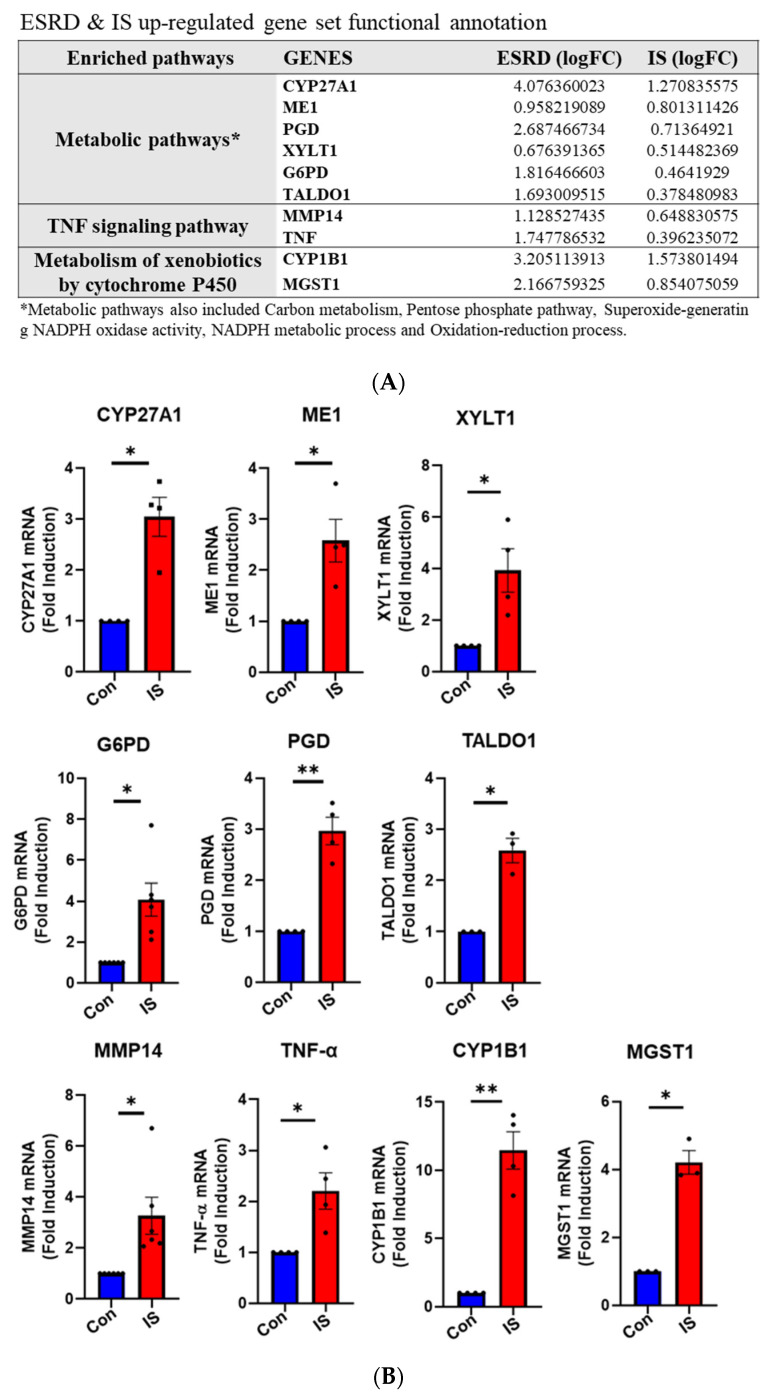
Functional annotation of upregulated gene sets and gene validation in ex vivo IS-stimulated monocytes. (**A**), 148 shared upregulated DEGs were analyzed via DAVID functional annotation and KEGG mapper, and validated major genes are listed. (**B**), Purified monocytes from independent HCs (*n* = 3–4) were treated with IS for 24 h, following RT-qPCR validation of genes listed in (**A**). * *p* < 0.05 and ** *p* < 0.01 compared to control group by two-tailed paired *t*-test.

By analyzing shared DEGs between ESRD patient-derived monocytes and IS-treated monocytes we have shown that genes in metabolic and AhR pathways are similarly regulated and interconnected. Next, we performed functional and GO annotation analyses using DAVID functional annotation and KEGG mapping to confirm distinct enrichment of a range of genes in several GO categories based on common upregulated DEGs in IS-treated monocytes and ESRD patient monocytes. As described in STRING and ClueGo analysis (Figure 4 and Figure S2), metabolic pathways including the carbon pathway, PPP, and glutathione metabolism (19 genes), phagosome (seven genes), cytokine-cytokine receptor interaction (six genes), NF-κB signaling (four genes), TNF signaling (two genes), and metabolism of xenobiotics by cytochrome p450 (two genes) were enriched GO terms in DEGs common between IS-stimulated and ESRD patient-derived monocytes (Figure 5A, and Table S4). Major genes associated with these pathways are listed in Figure 5A. To confirm expression of selected DEGs, we examined mRNA expression of a total of 10 genes listed in Figure 5A using IS-treated monocytes, which were obtained from independent HCs. The mRNA expression of CYP27A1, ME1, PGD, XYLT1, G6PD, and TALDO1 (associated with metabolic pathways) was significantly upregulated compared to control monocytes (Figure 5B). Especially, the mRNA expression of PPP-related genes such as G6PD, PGD, and TALDO1, were increased by IS treatment in a dose-dependent manner (Figure S3A–C) and as early as 4 h after stimulation (Figure S3D–F). TNF-α and MMP14 (related to TNF signaling) and CYP1B1 and MGST1 (involved in xenobiotic metabolism) were markedly increased by IS treatment of monocytes. These findings demonstrate that IS is responsible for changes in expression of genes in the metabolic and inflammatory-related pathways, which are the major pathways altered in ESRD patient-derived monocytes.

## 3. Discussion

In the present study, we found that IS, but not PCS, leads to marked alteration of gene expression in monocytes from HCs and among these differentially expressed genes (DEGs), 287 were shared with DEGs in ESRD patient-derived monocytes. Principle enriched pathways and interaction network analysis of DEGs demonstrated that several metabolic pathways, such as the pentose phosphate pathway, are primarily influenced by IS-rich uremic milieu in ESRD patient-derived monocytes. These findings were validated in ex vivo IS-stimulated monocytes where increased mRNA expression of Glucose-6-phosphate dehydrogenase (G6PD), phospho-gluconate dehydrogenase (PGD), trans-aldolase 1 (TALDO1), CYP1B1, and microsomal glutathione S-transferase 1 (MGST1) was found. Our findings suggest that IS contributes to altered metabolic pathways in monocytes of ESRD patients, and thus, these altered genes and pathways may be critical targets for modulating inflammatory responses of monocytes in CKD patients.

Cardiovascular disease (CVD) is a highly common complication and the major cause of death in patients with end-stage renal disease (ESRD) [6]. Several mechanisms have been proposed for the heightened risk for CVD in CKD, including infiltration of monocytes into arteries, increased cytokine production, and endothelial dysfunction [15,23]. Among over 100 uremic toxins identified, IS and PCS have been extensively investigated as the main uremic toxins involved in the progression of CVD [27,28,29]. Both IS and PCS are difficult to eliminate via classical dialysis approaches due to their strong protein-binding capabilities [27,30]. Moreover, both play a pivotal role in the pathogenesis of CVD through endothelial dysfunction, recruitment of leukocytes due to increased adhesion molecules such as VCAM-1, and reactive oxygen species (ROS) production [31,32]. The majority of studies have focused primarily on the adverse effects of IS and PCS on renal tissue, endothelial cells, and skeletal muscle cells [14,23,33]. Despite a key role of monocytes in the pathogenesis of CVD, the impact of IS and PCS on immune cells in patients with CKD remains elusive [13,16,17,34].

To identify potentially pathogenic biological pathways associated with major uremic toxins in monocytes of patients with CKD, we initially compared transcriptomic profiling between monocytes derived from ESRD patients and ex vivo IS- or PCS-stimulated monocytes from HCs. A total of 6630 genes were identified as differentially expressed genes (DEGs) in ESRD patient-derived monocytes compared to cells from HCs, whereas the number of DEGs was markedly smaller in IS- and PCS-treated monocytes (1599 and 270 genes, respectively; Figure 1 and Figure 2). Thus, this implies that a variety of uremic toxins besides IS and PCS can also directly or indirectly influence gene expression in monocytes during ESRD despite the crucial pathogenic roles of IS and PCS as major uremic toxins. Additionally, it is possible that chronic exposure to uremic toxin results in more profound changes in the gene profile of monocytes in ESRD patients. A comparative analysis of common DEGs using Venn diagrams suggests that alteration of gene expression in ESRD patient-derived monocytes is more associated with the effect of IS (287 genes) than with PCS (25 genes) (Figure 2). Our previous study demonstrated that, in contrast to IS, PCS does not induce production of TNF-α or IL-6 proinflammatory cytokines in human monocytes [16]. However, similar to IS, the concentration of PCS in serum of CKD patients has been reported to be associated with CVD complication in CKD [24] and a few studies have reported that PCS regulates the expression of TNF-α, MCP-1, and adhesion molecules, as well as ROS production in endothelial cells and macrophages, resulting in CVD in mice [13,35]. In contrast, consistent with our previous report, in the present study ex vivo PCS-stimulated human monocytes were not largely found to reflect the transcript profile of monocytes in patients with ESRD. Furthermore, IS led to mRNA induction of TNF-α and IL-1β in a dose-dependent manner (50~1000 μM) as depicted in previous report [16]. However, PCS had a minimal effect on mRNA induction of TNF-α and IL-1β at all doses (250~1000 μM) (Figure S4A,B). Therefore, we focused on the genes regulated by IS in ESRD patients.

Functional GSEA of monocytes from patients with ESRD identified the alteration of major metabolic pathways including glycolysis, oxidative phosphorylation, and fatty acid metabolism, as well as immune response-related pathways such as interferon-α and γ responses, the inflammatory response, and TNF-α signaling via NF-κB (Figure 3 and Table S3). We and others have demonstrated that IS elicits immune responses by its binding to AhR in monocytes/macrophages [36,37]. Thus, identification of immune response-related pathways in GSEA was expected. Furthermore, the notch signaling pathway was also identified in our GSEA (Figure S1B), which aligns with a recent study in which crosstalk between OATP2B1 and Dll4-Notch signaling in macrophages was found to mediate IS-induced vascular inflammation in CKD [17]. Of interest, a number of metabolic pathways were selected with higher NES (normal enrichment score) (Figure 3A and Table S3), suggesting that metabolic reprogramming of monocytes is induced when they are exposed to uremic milieu and is perhaps linked to inflammatory regulation in monocytes of ESRD patients. An increasing body of evidence demonstrates that activated monocytes/macrophages strictly modulate their metabolic programs to meet the demands of participation in immune responses such as cytokine production, and that metabolites play important roles in epigenetic modifications [38,39,40]. Much focus recently has been on understanding the interconnection between metabolic reprogramming and immune function for discovering important therapeutic targets in many inflammatory disorders [40,41,42,43]. In diabetic CKD patients, systemic metabolic failure induced by obesity, insulin resistance, and β-cell dysfunction exacerbates the progression of renal disease [44]. IS-mediated metabolic alterations in skeletal muscle also induce uremic sarcopenia in CKD [45]. However, little is known about metabolic alteration of immune cells in ESRD patients.

Network analysis using STRING software revealed five predicted protein-protein interaction (PPI) networks from commonly upregulated DEGs between ESRD patient-derived monocytes and IS-stimulated monocytes under stringent conditions. Among 35 selected protein nodes, TNF-α, CXCL8, CCR1, and C3 were found to be interconnected and each of their networks was further expanded by additional proteins (Figure 4A). Several studies have demonstrated that an increase in TNF-α in the uremic milieu is involved in renal dysfunction [46]. TNF-α is also considered an important pro-atherogenic cytokine in CVD [47]. In addition, IS is a potent endogenous ligand for AhR, and IS-stimulation of monocytes/macrophages results in increased production of TNF-α through a complicated regulation mechanism between AhR, NF-κB, and SOCS2 [16,36]. Chemokines and their receptors regulate migration of leukocytes under normal and inflammatory conditions. CXCL8 is secreted by activated monocytes and potently attracts neutrophils to inflamed sites [48], whereas expression of CCR1 on monocytes is increased during macrophage differentiation and is also critical for their recruitment to sites of inflammation [49]. Although most components of the complement system are produced by hepatocytes, a variety of innate cells, including stimulated monocytes and macrophages, are extrahepatic producers of complement proteins, such as C3, suggesting its local or intracellular role in immune responses [50]. Given the pivotal role of inflammatory monocytes/macrophages for pathogenesis of CVD, IS-mediated alteration of gene expression in monocytes may favor their infiltration into vessels, differentiation into macrophages, and local activation in CKD [51,52].

In addition, the interaction network generated by STRING and ClueGo analysis revealed the pentose biosynthetic process as a commonly enriched pathway (Figure 4A, red circle and Figure 4B, blue rectangle). Our confirmatory qPCR assay showed that the expression of G6PD, PGD, and TALDO1 genes of the pentose biosynthetic process is significantly increased in IS-treated monocytes from independent HCs (Figure 5). PPP is a metabolic pathway parallel to glycolysis. However, PPP does not produce or use energy in the form of ATP [53]. It has been demonstrated that LPS-stimulated macrophages upregulate the activity of PPP, supplying precursors for nucleotide synthesis and nicotinamide adenine dinucleotide phosphate (NADPH), which is used for reactive oxygen species (ROS) production by NADPH oxidase, fatty acid synthesis, and anti-oxidant cellular defense [54]. Sato et al. recently reported that IS induces metabolic alterations, such as upregulation of glycolysis with PPP acceleration, as an anti-oxidative stress response in skeletal muscle cells. This alteration induces uremic sarcopenia through downregulation of the TCA cycle, mitochondrial damage, and ATP shortage [45], suggesting that IS has the ability to alter metabolic pathways. In agreement with our findings, a single-cell analysis of PBMCs in ESRD illustrated major changes in the metabolic pathways of CD14^+^ monocytes [18].

The present study has several limitations. First, all microarray and ex vivo experiments were conducted in the culture media supplemented with 10% FBS but not with human albumin despite IS and PCS being mainly bind to albumin in vivo [55]. Thus, it is possible that the concentration of IS is at supraphysiological level. Additionally, no potassium adjustments were performed for the controls even though IS is a potassium salt [56]. These points must be carefully considered in understanding our findings. Second, circulating monocytes of ESRD patients are chronically exposed to various uremic toxins, while our microarray study was designed to induce an acute reaction to exposure to a specific uremic toxin such as IS and PCS for a short period of 24 h. Thus, we cannot rule out the possibility that a part of the gene expression changes affected by IS was identified. Given a short circulating lifespan (mean 1.0 ± 0.26 day) of classical CD14^+^ monocytes in vivo [57] and a technical difficulty of maintaining monocytes without differentiation into macrophages for more than at least 24 h ex vivo, IS-mediated changes in gene expression in peripheral monocytes would have been evaluated in the present study. Future studies are required to investigate the long-term effect of IS on the differentiation program and gene expression profiling of macrophages utilizing transcriptome analysis. Finally, the concentrations of IS and PCS differed in initial microarray experiments. In fact, the concentrations we used were determined based on our previously published report, showing that IS has more a potent effect on induction of TNF-α mRNA in monocytes compared with PCS [16]. In the present study, we also found that IS led to mRNA induction of TNF-α and IL-1β in a dose-dependent manner (50~1000 μM), whereas PCS had a minimal effect on mRNA induction of TNF-α and IL-1β at all doses (250~1000 μM) (Figure S4A,B). It would have been better to use the same concentration of IS and PCS in the microarray study. However, it is likely that the transcriptome analysis of monocytes treated even with the concentration of IS and PCS we used in this study is meaningful based on the above reasons.

In summary, the current study provides new insight into IS-mediated alteration of gene expression and biological pathways in ESRD patient-derived monocytes via analysis of their transcriptome by microarray. IS primarily contributes to metabolic reprograming including upregulated activity of PPP and changes in AhR and inflammatory signaling in monocytes of ESRD patients. Our data suggest that these altered genes and pathways may be critical targets for modulating inflammatory responses of monocytes, which are key players in the pathogenesis of CVD in CKD patients.

## 4. Materials and Methods

### 4.1. Peripheral Blood Mononuclear Cell (PBMC) Isolation

The study protocols were reviewed and approved by the institutional review board of Seoul National University Hospital and Severance Hospital, Korea. Peripheral blood of ESRD patients and healthy controls (HCs) was drawn after obtaining written, informed consent. The methods were performed in accordance with the approved guidelines (IRB No.1403-049-564 for Seoul National University Hospital, Date: 31 March 2014 and IRB No. 4-2013-0581 for Severance Hospital, Date: 10 October 2013). Mononuclear cells were isolated from peripheral blood by density gradient centrifugation using Bicoll separating solution (BIOCHROM Inc, Cambridge, UK).

### 4.2. Sample Preparation for Microarray

Isolation of monocytes from PBMCs of three HCs and three ESRD patients between the ages of 50 and 75 were performed through a positive selection using anti-CD14 magnetic beads (Miltenyi Biotec, Bergish, Gladbach, Germany). The demographic characteristics of ESRD patients enrolled in microarray analysis are summarized in Table S1. Monocytes for ex vivo approaches were separated from PBMC of three healthy subjects through negative selection using pan-monocyte microbeads (Miltenyi Biotec Inc, Auburn, CA, USA), then further treated with IS (1000 μM) or PCS (500 μM) for 24 h in RPMI 1640 medium supplemented with 10% fetal bovine serum (FBS), 100 units/mL penicillin, 100 μg/mL streptomycin, and 2 mM L-glutamine. Total RNA from all samples was prepared using an RNA purification kit (Qiagen) and checked for integrity and purity by OD 260/280 ratio on the Agilent 2100 Bioanalyzer (Agilent Technologies, Santa Clara, CA, USA).

### 4.3. Microarray Gene Expression Analysis

The Affymetrix Whole transcript (WT) Expression array process was conducted with reference to the instructions provided by the manufacturer (GeneChip Whole Transcript PLUS reagent Kit, ThermoFisher scientific, Waltham, MA, USA). cDNAs were synthesized using the GeneChip WT Amplification kit, followed by cDNA fragmentation. Consecutively, fragmented cDNAs were biotin-labelled with terminal deoxynucleotidyl tranferase (TdT) using the GeneChip WT Terminal labelling kit. Approximately 5.5 μg of labelled DNA targets was hybridized into the Affymetrix GeneChip Human 2.0 ST Array at 45 °C for 16 h. Hybridized arrays were then washed and stained on the GeneChip Fluidics Station 450 and scanned on a GCS3000 Scanner (Affymetrix, ThermoFisher scientific, Waltham, MA, USA). Measured signal values were computed using the Affymetrix^®^ GeneChip™ Command Console software. Raw data of microarray have been deposited in the NCBI Gene Expression Omnibus (accession code GSE155325 and GSE155326).

### 4.4. Data Preparation and Descriptive Statistics

The microarray represents approximately 40,716 human genes. Raw data were obtained automatically with reference to the Affymetrix data extraction protocol using the software provided by Affymetrix GeneChip^®^ Command Console^®^ Software (AGCC). Data were summarized and normalized with Robust Multi-array Average (RMA) method provided by Affymetrix^®^ Expression Console™ Software (EC) (ThermoFisher scientific, Waltham, MA, USA) or the R-package Oligo (ver. 1.52.0) [58] and limma (ver. 3.44.3) [59]. We obtained the data using gene-level RMA analysis and differentially expressed gene (DEG) analysis. The comparative analysis was carried out using *T*-test. Moreover, false discovery rate (FDR) was controlled by adjusting *p*-value via Benjamini-Hochberg algorithm. For DEG sets, hierarchical cluster analysis was conducted using the complete linkage with Euclidean distance as a measure of similarities between DEG sets. All statistical tests and visualization of DEGs were conducted using R statistical language v. 3.6.2. (www.r-project.org) [60].

### 4.5. Gene Expression Profiling and Analysis

Genes were selected based on *p*-value of 0.05 or less and corrected through Benjamin and Hochberg multiple testing (a false discovery rate) of 25% (FDR = 0.25). Selected data were then applied to hierarchical cluster analysis to display basal and luminal differences. These data were further filtered according to gene expression levels with a log2 fold change of <−0.3 and >0.3. Collected microarray data of common genes between ESRD and IS were visualized with heatmap using the pheatmap R package (https://rdrr.io/cran/pheatmap) [61].

### 4.6. Gene Set Enrichment Analysis

Gene Set Enrichment Analysis (GSEA) [26,62] was used to examine the significantly-enriched pathways with the normalized microarray dataset of the ESRD/HC group to various gene sets in the GSEA Molecular Signatures Database (MsigDB). All transcripts within annotated genes (~31,727 features in total) regarding expression values were uploaded to a locally-installed GSEA tool and compared with the catalog H (50 human gene sets) [63]. The reported GSEA outputs were filtered based on a normalized enrichment score (NES) > 1.3.

### 4.7. GO and Protein Pathway Analysis

Protein pathways associated with commonly expressed genes were examined by STRING analysis (https://string-db.org/). Protein–protein, direct (physical) or indirect (functional) interactions (highest confidence: 0.900) were critically assessed based on STRING data base. The intensity of edges reflects the degree of interaction score of gene/protein interaction. Additionally, each group of proteins associated with particular biological pathways are presented by different colors representing the network nodes.

Furthermore, Gene-functionality grouped networks were visualized by Cytoscape software (https://cytoscape.org/index.html). Two plug-ins of Cytoscape, ClueGO (ver. 2.5.7) [64] and CluePedia (ver. 1.5.7) [65] were used in the present study. The results of PPI network analysis are illustrated by large clusters of genes which were classified into different functional groups on the basis of GO, KEGG, Wiki Pathways, and Reactome.

### 4.8. Gene Functional Annotation and GO Annotation

Gene functional annotation and GO annotation were conducted by importing significant gene sets into the Gene Functional Annotation Tool available at the DAVID (http://david.abcc.ncifcrf.gov/) and KEGG Mapper websites (https://www.genome.jp/kegg/tool/map_pathway2.html) (*p*-value < 0.05).

### 4.9. Validation Microarray Genes-Quantitative RT-PCR

To validate common DEGs in ex vivo monocytes, PBMCs of HCs were separated and CD4 positive monocytic cells were sorted. Then, these cells were treated with IS for 24 hr. Total RNA of all samples was separated by using RNA purification kit (Macherey-Nagel GmbH & Co. KG, Düren, Germany) followed by cDNA synthesis (Bio-line, London, UK). Subsequently, real-time quantitative RT-PCR was performed through CFX system (Bio-Rad, Hercules, CA, USA) with SensiFAST SYBR^®^ Lo-ROX (Bio-line, London, UK). The primers used in this investigation were shown in Table S5. Normalization of gene expression levels was based on the expression of ACTINB. In addition, the comparative CT method (ΔΔCT) was used for quantification of gene expression.

For data analysis, two-tailed paired student’s *t*-test were conducted using Graph Pad Prism 8 (GraphPad Software, La Jolla, CA, USA) and Microsoft Excel 2013. *p* < 0.05 was considered statistically significant.

## Figures and Tables

**Table 1:** Upregulated and downregulated DEGs (50 each) common in ESRD patient-derived monocytes and ex vivo IS-treated monocytes. Common DEGs are listed according to a log2 fold change (*p* < 0.05, FDR < 0.25).

	Probe ID	Symbol	Gene Name	Fold Change (log2)	
				ESRD	IS
Top 50 Up-Regulated Genes	16967771	CXCL8	C-X-C motif chemokine ligand 8	6.5945	0.3171
	17000793	CD14	CD14 molecule	6.1733	0.3099
	17058719	NCF1C	neutrophil cytosolic factor 1C pseudogene	4.9855	0.7591
	16716590	MYOF	myoferlin	4.7301	0.3701
	16857736	MCEMP1	mast cell expressed membrane protein 1	4.5776	0.6207
	16854486	DSC2	desmocollin 2	4.4608	0.3290
	16952874	CCR1	C-C motif chemokine receptor 1	4.3715	0.3022
	16869666	ADGRE3	adhesion G protein-coupled receptor E3	4.2049	0.7200
	16891082	CYP27A1	cytochrome P450 family 27 subfamily A member 1	4.0764	1.2708
	16709333	TCF7L2	transcription factor 7 like 2	3.9883	0.9611
	16684056	FGR	FGR proto-oncogene, Src family tyrosine kinase	3.8943	0.4057
	16768297	DUSP6	dual specificity phosphatase 6	3.8745	0.3807
	17086496	DAPK1	death associated protein kinase 1	3.8445	0.4779
	17059955	PDK4	pyruvate dehydrogenase kinase 4	3.5273	0.4756
	17096030	FBP1	fructose-bisphosphatase 1	3.4529	0.4545
	17046911	NCF1B	neutrophil cytosolic factor 1B pseudogene	3.3574	1.0349
	16896561	CYP1B1	cytochrome P450 family 1 subfamily B member 1	3.2051	1.5738
	16684144	PTAFR	platelet activating factor receptor	3.1942	0.3886
	17000724	HBEGF	heparin binding EGF like growth factor	2.8582	0.8480
	17044442	SNX10	sorting nexin 10	2.8334	0.3116
	16732985	SLC37A2	solute carrier family 37 member 2	2.7402	0.3424
	16658864	PGD	phospho-gluconate dehydrogenase	2.6875	0.7136
	17111711	VSIG4	V-set and immunoglobulin domain containing 4	2.6333	0.6154
	16805474	ARRDC4	arrestin domain containing 4	2.5744	0.3588
	16777685	FLT3	fms related tyrosine kinase 3	2.5176	0.4135
	17053697	LOC644090	uncharacterized LOC644090	2.4605	0.6179
	16991125	NDST1	N-deacetylase and N-sulfotransferase 1	2.3802	0.3088
	16669196	CD101	CD101 molecule	2.2198	0.5474
	17063722	CLEC5A	C-type lectin domain containing 5A	2.2079	0.6715
	16875723	TMEM150B	transmembrane protein 150B	2.1721	0.3627
	16748788	MGST1	microsomal glutathione S-transferase 1	2.1668	0.8541
	16867784	C3	complement C3	2.1475	0.5078
	17121820	USP32	ubiquitin specific peptidase 32	2.1419	0.3092
	16929920	H1F0	H1 histone family member 0	2.0673	0.3007
	16848070	SLC16A6	solute carrier family 16 member 6	2.0182	0.4231
	16839524	MIR22HG	MIR22 host gene	2.0173	0.4588
	16774427	LACC1	laccase domain containing 1	1.9373	0.7574
	16729298	ACER3	alkaline ceramidase 3	1.9074	0.4178
	16894335	ADAM17	ADAM metallopeptidase domain 17	1.9073	0.3308
	17026267	TNF	tumor necrosis factor	1.8353	0.6968
	17013520	SASH1	SAM and SH3 domain containing 1	1.8301	0.6865
	17115636	G6PD	glucose-6-phosphate dehydrogenase	1.8165	0.4642
	16683445	FUCA1	alpha-L-fucosidase 1	1.7958	1.5007
	16876849	ASAP2	ArfGAP with SH3 domain, ankyrin repeat and PH domain 2	1.7922	0.3036
	16852871	SERPINB2	serpin family B member 2	1.7359	2.2714
	17058978	GSAP	gamma-secretase activating protein	1.7231	0.3256
	17071162	CPQ	carboxypeptidase Q	1.7211	0.3579
	16968447	GPAT3	glycerol-3-phosphate acyltransferase 3	1.7197	0.3522
	16720318	TALDO1	trans-aldolase 1	1.6930	0.3785
	16688929	GNG5	G protein subunit gamma 5	1.6846	0.3409
Top 50 Down-Regulated Genes	16781893	YME1L1	YME1 like 1 ATPase	−5.1212	−0.3594
	16798132	SNORD116-1	small nucleolar RNA, C/D box 116-1	−4.8499	−0.8151
	17074313	DEFA1B	defensin alpha 1B	−4.7542	−0.3063
	16761201	CD69	CD69 molecule	−4.1940	−0.3732
	16781830	TRAJ17	T cell receptor alpha joining 17	−3.9577	−0.3544
	16877297	TRIB2	tribbles pseudo-kinase 2	−3.6828	−0.3169
	16906571	STAT4	signal transducer and activator of transcription 4	−3.6785	−0.3412
	16798206	SNORD116-20	small nucleolar RNA, C/D box 116-20	−3.3709	−0.3708
	16672669	LY9	lymphocyte antigen 9	−3.2701	−0.3066
	16748205	CLEC2D	C-type lectin domain family 2 member D	−3.2426	−0.3819
	16761631	DUSP16	dual specificity phosphatase 16	−3.1818	−0.4038
	17110670	PIM2	Pim-2 proto-oncogene, serine/threonine kinase	−2.9802	−0.6422
	16912130	CST7	cystatin F	−2.5494	−0.3388
	17092767	MLLT3	MLLT3, super elongation complex subunit	−2.4775	−0.7344
	16720085	IFITM1	interferon induced transmembrane protein 1	−2.4475	−0.3137
	17056849	TRGV3	T cell receptor gamma variable 3	−2.4407	−0.8184
	17005858	HIST1H2AI	histone cluster 1 H2A family member i	−2.2799	−0.4418
	16995717	SNORD72	small nucleolar RNA, C/D box 72	−2.2604	−0.7078
	17016503	HIST1H3I	histone cluster 1 H3 family member i	−2.2455	−0.4036
	16764564	LIMA1	LIM domain and actin binding 1	−2.2305	−0.3310
	16934045	PIK3IP1	phosphoinositide-3-kinase interacting protein 1	−2.2022	−0.4481
	16900737	ANKRD36B	ankyrin repeat domain 36B	−2.0806	−0.3064
	17016386	HIST1H3D	histone cluster 1 H3 family member d	−2.0589	−0.3624
	17122454	ANKRD36	ankyrin repeat domain 36	−2.0333	−0.5181
	17121094	SRP54-AS1	SRP54 antisense RNA 1 (head to head)	−1.9509	−0.4420
	16756286	C12orf75	chromosome 12 open reading frame 75	−1.8645	−0.3758
	16798248	SNHG14	small nucleolar RNA host gene 14	−1.8228	−0.3124
	17122912	ANKRD20A5P	ankyrin repeat domain 20 family member A5, pseudogene	−1.8010	−0.3463
	16686060	SLC2A1	solute carrier family 2 member 1	−1.7904	−0.3997
	16968314	PRDM8	PR/SET domain 8	−1.7855	−0.5214
	17016383	HIST1H4D	histone cluster 1 H4 family member d	−1.7560	−0.3952
	16837296	ARHGAP27P2	Rho GTPase activating protein 27 pseudogene 2	−1.7467	−0.4159
	17005560	HIST1H4C	histone cluster 1 H4 family member c	−1.7216	−0.3526
	16849349	TNRC6C-AS1	TNRC6C antisense RNA 1	−1.6941	−0.6821
	17016360	HIST1H4B	histone cluster 1 H4 family member b	−1.6893	−0.5007
	17011708	SLC16A10	solute carrier family 16 member 10	−1.6648	−0.4770
	16780585	GPR18	G protein-coupled receptor 18	−1.6552	−0.4941
	17116745	P2RY8	P2Y receptor family member 8	−1.6285	−0.4951
	16987361	ARSK	arylsulfatase family member K	−1.6196	−0.3051
	16869324	PRDX2	peroxiredoxin 2	−1.6134	−0.4093
	16980528	LRBA	LPS responsive beige-like anchor protein	−1.6071	−0.3149
	16838300	TMC8	transmembrane channel like 8	−1.5657	−0.3583
	17100888	LOC102723630	uncharacterized LOC102723630	−1.5554	−0.4080
	16956983	CBLB	Cbl proto-oncogene B	−1.5203	−0.5545
	16930173	APOBEC3D	apolipoprotein B mRNA editing enzyme catalytic subunit 3D	−1.5054	−0.4054
	16985518	PIK3R1	phosphoinositide-3-kinase regulatory subunit 1	−1.5016	−0.5210
	17113606	septin 6	septin 6	−1.5012	−0.4799
	16993173	LOC728554	THO complex 3 pseudo-gene	−1.4969	−0.3166
	16889160	PLCL1	phospholipase C like 1 (inactive)	−1.4891	−0.3684
	17013197	PEX3	peroxisomal biogenesis factor 3	−1.4474	−0.3493

## Supplementary Materials

The following are available online at https://www.mdpi.com/2072-6651/12/10/621/s1, Figure S1: Gene set enrichment analysis (GSEA) plot of mTORC1 signaling (**A**) and notch signaling (**B**) of 35 enriched pathways analyzed using 6630 DEGs in monocytes of ESRD patients (*p* < 0.05, FDR < 0.25). Left panels are enrichment plots and right panels are heatmaps. Figure S2: GO Analysis of protein-protein interaction (PPI) networks for DEGs commonly downregulated in both ESRD patient-derived monocytes and ex vivo IS-stimulated monocytes of HCs. (**A**), STRING analysis derived from PPI networks for 139 selected downregulated monocyte genes. The network nodes represent proteins while edges represent protein interactions and strength. The average node degree was 0.673, and the local clustering coefficient average was 0.118. (**B**), ClueGO gene ontology analysis of 139 shared downregulated DEGs. Colors represent the statistical significance, where darker color indicates higher significance of GO group. Each node and edges show GO biomolecular pathways and the connectivity between each node, respectively. (**C**). Go biological process pie chart of ClueGo analysis (**B**) Enrichment score (%) indicates the upregulated DEGs involved in enriched Go biological processes. Figure S3: A dose-dependent effect of IS on G6PD, PGD, and TALDO1 mRNA expression (**A**–**C**) and time-kinetics on expression of these genes (**D**–**F**) in purified monocytes. Monocytes isolated from independent HCs (*n* = 4 or 5) were treated with IS at indicated concentration for 24 h (**A**–**C**) or in indicated times (**D**–**F**), followed by RT-qPCR. Figure S4: IS or PCS-induced mRNA expression of TNF-α (**A**) and IL-1β (**B**) in human monocytes. Monocytes isolated from independent HCs (*n* = 4 or 5) were treated with IS or PCS in the indicated concentration for 24 h, followed by RT-qPCR. Table S1: Baseline characteristics in study subjects. Table S2: Common upregulated (148) and downregulated (139) DEGs in monocytes of ESRD patients and IS-treated monocytes. Common DEGs are listed according to a log2 fold value (*p* < 0.05, FDR < 0.25). Table S3: Pathways (35) associated with DEGs in monocytes of patients with ESRD. These pathways are listed based on FWER *p*-value (*p* < 0.05, FDR < 0.25). Table S4: The enriched pathways and their associated genes in commonly upregulated DEGs in IS-treated monocytes and ESRD patient monocytes identified via analysis of a DAVID functional annotation and KEGG mapper. This list was ordered based on a log2 fold value of ESRD patients (*p* < 0.05, FDR < 0.25). Table S5: Primers for validated genes.
