# Supplementary material for: Indoxyl Sulfate-Mediated Metabolic Alteration of Transcriptome Signatures in Monocytes of Patients with End-Stage Renal Disease (ESRD)
Source: Toxins (Basel). 2020 Sep 28;12(10):621. doi: 10.3390/toxins12100621 (PMC7601745; doi:10.3390/toxins12100621)
Supplement: Supplementary file 1 [file toxins-12-00621-s001.pdf]

Supplementary Materials: Indoxyl Sulfate-Mediated Metabolic Alteration of Transcriptome Signatures in Monocytes of Patients with End-Stage Renal Disease (ESRD)

Hee Young Kim, Su Jeong Lee, Yuri Hwang, Ga Hye Lee, Chae Eun Yoon, Hyeon Chang Kim, Tae-Hyun Yoo and Won-Woo Lee

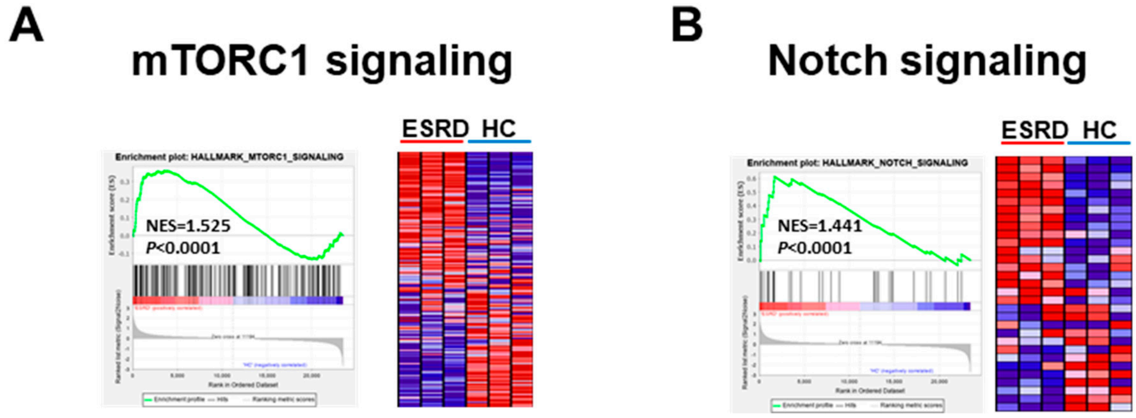

Figure S1. Gene set enrichment analysis (GSEA) plot of mTORC1 signaling (**A**) and notch signaling (**B**) of 35 enriched pathways analyzed using 6630 DEGs in monocytes of ESRD patients ($p < 0.05$, FDR < 0.25). Left panels are enrichment plots and right panels are heatmaps.

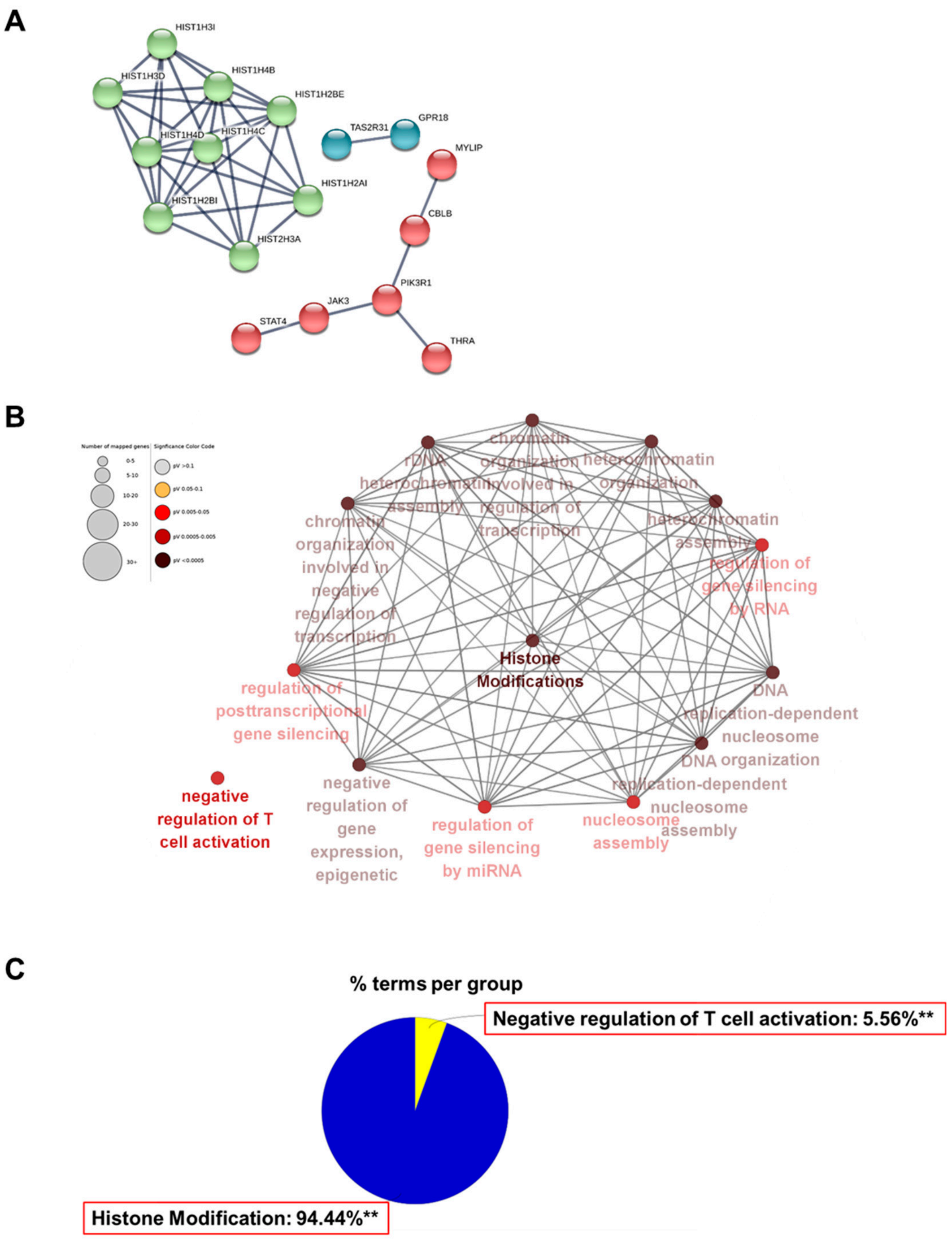

Figure S2. GO Analysis of protein-protein interaction (PPI) networks for DEGs commonly downregulated in both ESRD patient-derived monocytes and ex vivo IS-stimulated monocytes of HCs. (**A**), STRING analysis derived from PPI networks for 139 selected downregulated monocyte genes. The network nodes represent proteins while edges represent protein interactions and strength. The average node degree was 0.673, and the local clustering coefficient average was 0.118. (**B**), ClueGO gene ontology analysis of 139 shared downregulated DEGs. Colors represent the statistical significance, where darker color indicates higher significance of GO group. Each node and edges show GO biomolecular pathways and the connectivity between each node, respectively. (**C**). Go biological process pie chart of ClueGo analysis (**B**) Enrichment score (%) indicates the upregulated DEGs involved in enriched Go biological processes. ** $p < 0.01$ indicates the significant terms in the group.

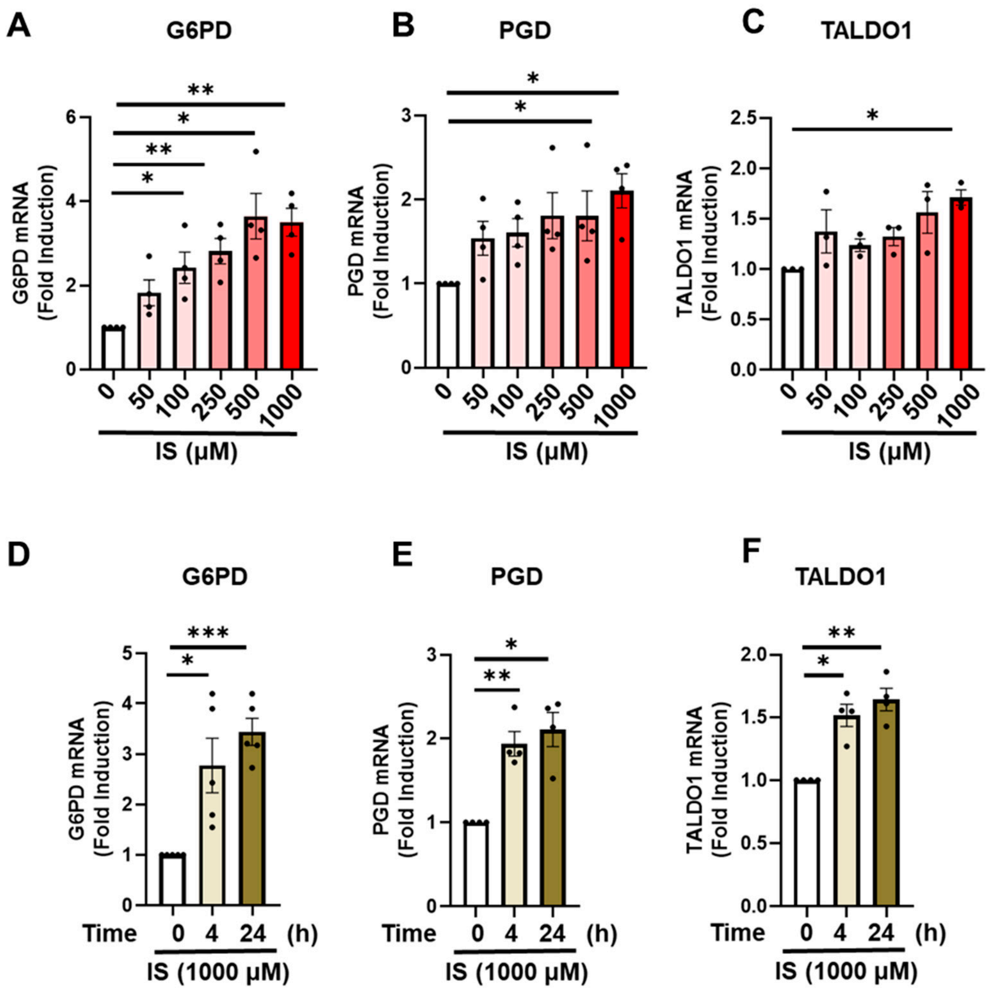

Figure S3. A dose-dependent effect of IS on G6PD, PGD, and TALDO1 mRNA expression (**A**–**C**) and time-kinetics on expression of these genes (**D**–**F**) in purified monocytes. Monocytes isolated from independent HCs (n = 4 or 5) were treated with IS at indicated concentration for 24 hr (**A**–**C**) or in indicated times (**D**–**F**), followed by RT-qPCR. * $p < 0.05$ and ** $p < 0.01$ compared to control group by two-tailed paired t-test.

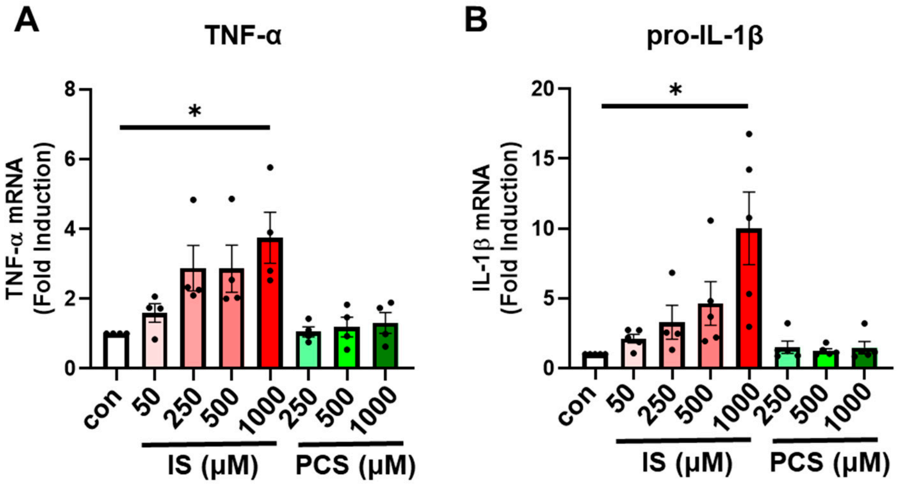

Figure S4. IS or PCS-induced mRNA expression of TNF-α (**A**) and IL-1β (**B**) in human monocytes. Monocytes isolated from independent HCs (n = 4 or 5) were treated with IS or PCS in the indicated concentration for 24 hr, followed by RT-qPCR. * $p < 0.05$ compared to control group by two-tailed paired t-test.

Table S1. Baseline characteristics in study subjects.

	Case 1	Case 2	Case 3
Age, year	74	61	54
Sex	M	M	F
Body mass index, kg/m^2	1.54	1.84	1.67
Dialysis duration (yr)	12.1	2.6	16.8
Cause of ESRD	Hypertension	Chronic glomerulonephritis	Diabetes mellitus
WBC, /mm^3	6770	6480	5510
Hemoglobin, g/L	10.3	11.0	9.4
Albumin, g/L	3.7	4.6	3.7
hs-CRP, mg/L	11.1	2.5	0.4

ESRD, end stage renal disease.

Table S2. Common upregulated (148) and downregulated (139) DEGs in monocytes of ESRD patients and IS-treated monocytes. Common DEGs are listed according to a log2 fold value ($p < 0.05$, FDR < 0.25).

Upregulated DEGs				Downregulated DEGs			
Probe ID	Symbol	Fold Change (log2)		Probe ID	Symbol	Fold Change (log2)	
		ESRD	IS			ESRD	IS
16967771	CXCL8	6.594	0.317	16781893	YME1L1	−5.121	−0.359
17000793	CD14	6.173	0.310	16798132	SNORD116-1	−4.850	−0.815
17058719	NCF1C	4.986	0.759	17074313	DEFA1B	−4.754	−0.306
16716590	MYOF	4.730	0.370	16761201	CD69	−4.194	−0.373
16857736	MCEMP1	4.578	0.621	16781830	TRAJ17	−3.958	−0.354
16854486	DSC2	4.461	0.329	16877297	TRIB2	−3.683	−0.317
16952874	CCR1	4.372	0.302	16906571	STAT4	−3.678	−0.341
16869666	ADGRE3	4.205	0.720	16798206	SNORD116-20	−3.371	−0.371
16891082	CYP27A1	4.076	1.271	16672669	LY9	−3.270	−0.307
16709333	TCF7L2	3.988	0.961	16748205	CLEC2D	−3.243	−0.382
16684056	FGR	3.894	0.406	16761631	DUSP16	−3.182	−0.404
16768297	DUSP6	3.875	0.381	17110670	PIM2	−2.980	−0.642
17086496	DAPK1	3.845	0.478	16912130	CST7	−2.549	−0.339
17059955	PDK4	3.527	0.476	17092767	MLLT3	−2.478	−0.734
17096030	FBP1	3.453	0.455	16720085	IFITM1	−2.447	−0.314
17046911	NCF1B	3.357	1.035	17056849	TRGV3	−2.441	−0.818
16896561	CYP1B1	3.205	1.574	17005858	HIST1H2AI	−2.280	−0.442
16684144	PTAFR	3.194	0.389	16995717	SNORD72	−2.260	−0.708
17000724	HBEGF	2.858	0.848	17016503	HIST1H3I	−2.246	−0.404
17044442	SNX10	2.833	0.312	16764564	LIMA1	−2.231	−0.331
16732985	SLC37A2	2.740	0.342	16934045	PIK3IP1	−2.202	−0.448
16658864	PGD	2.687	0.714	16900737	ANKRD36B	−2.081	−0.306
17111711	VSIG4	2.633	0.615	17016386	HIST1H3D	−2.059	−0.362
16805474	ARRDC4	2.574	0.359	17122454	ANKRD36	−2.033	−0.518
16777685	FLT3	2.518	0.414	17121094	SRP54-AS1	−1.951	−0.442
17053697	LOC644090	2.460	0.618	16756286	C12orf75	−1.865	−0.376
16991125	NDST1	2.380	0.309	16798248	SNHG14	−1.823	−0.312
16669196	CD101	2.220	0.547	17122912	ANKRD20A5P	−1.801	−0.346
17063722	CLEC5A	2.208	0.672	16686060	SLC2A1	−1.790	−0.400
16875723	TMEM150B	2.172	0.363	16968314	PRDM8	−1.785	−0.521
16748788	MGST1	2.167	0.854	17016383	HIST1H4D	−1.756	−0.395
16867784	C3	2.148	0.508	16837296	ARHGAP27P2	−1.747	−0.416
17121820	USP32	2.142	0.309	17005560	HIST1H4C	−1.722	−0.353
16929920	H1F0	2.067	0.301	16849349	TNRC6C-AS1	−1.694	−0.682
16848070	SLC16A6	2.018	0.423	17016360	HIST1H4B	−1.689	−0.501
16839524	MIR22HG	2.017	0.459	17011708	SLC16A10	−1.665	−0.477
16774427	LACC1	1.937	0.757	16780585	GPR18	−1.655	−0.494

16729298	ACER3	1.907	0.418	17116745	P2RY8	−1.629	−0.495
16894335	ADAM17	1.907	0.331	16987361	ARSK	−1.620	−0.305
17026267	TNF	1.835	0.697	16869324	PRDX2	−1.613	−0.409
17013520	SASH1	1.830	0.686	16980528	LRBA	−1.607	−0.315
17115636	G6PD	1.816	0.464	16838300	TMC8	−1.566	−0.358
16683445	FUCA1	1.796	1.501	17100888	LOC102723630	−1.555	−0.408
16876849	ASAP2	1.792	0.304	16956983	CBLB	−1.520	−0.555
16852871	SERPINB2	1.736	2.271	16930173	APOBEC3D	−1.505	−0.405
17058978	GSAP	1.723	0.326	16985518	PIK3R1	−1.502	−0.521
17071162	CPQ	1.721	0.358	17113606	septin 6	−1.501	−0.480
16968447	GPAT3	1.720	0.352	16993173	LOC728554	−1.497	−0.317
16720318	TALDO1	1.693	0.378	16889160	PLCL1	−1.489	−0.368
16688929	GNG5	1.685	0.341	17013197	PEX3	−1.447	−0.349
16748590	EMP1	1.667	0.527	16692805	GOLPH3L	−1.431	−0.410
16725783	BEST1	1.659	0.333	16791898	CFL2	−1.408	−0.436
17042681	FAM20C	1.617	0.850	16886200	ARHGAP15	−1.405	−0.453
17058826	HIP1	1.612	0.533	17083357	CD274	−1.384	−0.542
16707673	TBC1D12	1.599	0.644	17067410	FZD3	−1.381	−0.382
17126024	TRIM25	1.590	0.347	17068014	DDHD2	−1.348	−0.374
16863307	VASP	1.573	0.306	16850656	DLGAP1-AS1	−1.332	−0.427
16939815	ABHD5	1.572	0.306	16718106	STN1	−1.311	−0.358
16986700	ZFYVE16	1.548	0.302	16978036	HPGDS	−1.291	−0.304
16822728	CLCN7	1.544	0.463	17123270	TRANK1	−1.275	−0.435
17099436	GBGT1	1.514	0.308	16881687	WDR54	−1.268	−0.501
16868000	CD209	1.479	0.435	16970673	JADE1	−1.255	−0.318
17007673	UHRF1BP1	1.473	0.309	17101739	CA5B	−1.249	−0.503
16891042	TTLL4	1.469	0.567	17120696	LOC374443	−1.239	−0.442
16925328	KCNE1	1.468	0.982	16987298	SLF1	−1.238	−0.406
16867344	LRG1	1.440	0.799	16938378	NR1D2	−1.179	−0.349
16879232	CYP1B1-AS1	1.413	0.869	16768438	BTG1	−1.172	−0.332
17086540	CTSL	1.346	0.469	16842850	DHRS13	−1.166	−0.413
17070456	CA2	1.294	0.378	17005578	HIST1H2BE	−1.115	−0.326
16733473	ST14	1.278	0.354	17005603	HIST1H2BI	−1.108	−0.662
16773759	FRY	1.278	0.340	16906440	OSGEPL1	−1.102	−0.407
16712531	ARHGAP21	1.272	0.433	16869291	SNORD41	−1.094	−0.336
16672478	SLAMF8	1.272	0.650	16691090	PTPN22	−1.063	−0.420
17066961	ADAMDEC1	1.272	0.395	16689400	GBP5	−1.056	−1.028
17021585	UBE2J1	1.264	0.442	17121072	FLJ22447	−1.028	−0.378
17126000	ATF5	1.241	0.313	16762573	INTS13	−1.026	−0.399
16852997	SOCS6	1.231	0.444	16669821	LIX1L	−1.021	−0.393
16804539	ABHD2	1.223	0.328	16860123	ZNF486	−1.011	−0.443
16800680	SQOR	1.206	0.345	16676183	ATP2B4	−1.011	−0.968
17115868	IL3RA	1.203	0.764	16833965	THRA	−1.010	−0.319
17090042	C9orf106	1.184	0.304	16957095	CD47	−1.007	−0.474
16942576	MITF	1.171	0.370	16870274	JAK3	−1.005	−0.428
17081162	ASAP1	1.159	0.352	17058121	ZNF680	−0.989	−0.499
16760668	LPCAT3	1.151	0.325	16967031	PAICS	−0.984	−0.385
17005042	JARID2	1.140	0.453	16737102	TPT1-AS1	−0.978	−0.306
17053769	LINC01003	1.134	0.337	16747570	ENO2	−0.970	−0.511
16782187	MMP14	1.129	0.649	16709822	GRK5	−0.957	−0.367
16700515	SIPA1L2	1.105	0.564	16676999	TRAF3IP3	−0.952	−0.542
17096728	ABCA1	1.088	0.307	16936137	CERK	−0.917	−0.356
16851933	GALNT1	1.087	0.343	17005077	MYLIP	−0.894	−0.347
17058142	ZNF117	1.035	0.324	17001800	ANXA6	−0.887	−0.419
16919158	TGM2	1.033	0.943	16900293	ANKRD36C	−0.863	−0.576
17087790	SLC44A1	1.032	0.523	16759635	ZNF84	−0.860	−0.414

16974250	SLC2A9	1.030	0.361	17005655	BTN3A1	−0.860	−0.428
17086048	GCNT1	1.018	0.405	17042844	GPR146	−0.854	−0.430
16718493	LOC105378488	1.012	0.424	16702047	NET1	−0.813	−0.419
16725670	DAGLA	1.004	0.555	16692724	ANP32E	−0.810	−0.383
16696187	F5	0.999	0.606	16978417	SLC39A8	−0.793	−0.556
16826230	NETO2	0.995	0.357	17057842	LOC102723533	−0.787	−0.615
16971966	FNIP2	0.995	0.613	16703431	FAM238A	−0.784	−0.595
16672333	CD1E	0.970	0.410	16670387	HIST2H3A	−0.780	−0.304
16751401	ACVR1B	0.961	0.317	16864975	ZNF813	−0.772	−0.399
17021217	ME1	0.958	0.801	16748888	PLEKHA5	−0.752	−0.310
17047225	NCF1	0.957	0.802	16803807	IL16	−0.740	−0.308
16980918	CTSO	0.949	0.359	16954478	NPRL2	−0.740	−0.346
16694322	RIT1	0.947	0.353	16885290	GYPC	−0.716	−0.354
16916352	SRXN1	0.940	0.513	16922183	C21orf62-AS1	−0.702	−0.303
17101447	HCCS	0.882	0.328	16884297	LOC105373547	−0.694	−0.345
16839412	PITPNA	0.877	0.304	16914741	SNORD12C	−0.694	−0.432
16714776	EGR2	0.874	0.722	16870759	ZNF682	−0.693	−0.378
16888512	ITGAV	0.842	0.472	16817349	NPIPB8	−0.687	−0.309
16880712	SLC1A4	0.827	0.314	17120318	PARP8	−0.649	−0.431
16867074	MFSD12	0.817	0.314	17082851	ZNF251	−0.646	−0.374
16713187	NRP1	0.754	0.453	16981516	LOC105377540	−0.643	−0.626
17005234	E2F3	0.746	0.347	17015240	ECI2	−0.639	−0.301
16702836	MRC1	0.743	1.184	16977502	PLAC8	−0.634	−0.380
16839769	P2RX5	0.698	0.317	16661051	PDIK1L	−0.621	−0.303
17114272	GPC4	0.683	0.560	17016221	RIPOR2	−0.620	−0.319
16669180	PTGFRN	0.682	0.770	17117467	GOLGA7B	−0.618	−0.468
16824352	XYLT1	0.676	0.514	16713335	ZNF248	−0.614	−0.338
16901593	septin 10	0.674	0.420	16738395	OR5AP2	−0.611	−0.348
16728788	P2RY6	0.656	0.379	16780236	TGDS	−0.606	−0.323
16739026	CYB561A3	0.655	0.391	16775883	MBNL2	−0.590	−0.722
16770915	WSB2	0.645	0.343	16720720	KRTAP5-5	−0.568	−0.334
17000465	ETF1	0.635	0.329	17005955	ZKSCAN3	−0.538	−0.469
16999769	LOC102723741	0.634	0.479	17053384	GIMAP4	−0.531	−0.529
16911482	LOC105372535	0.633	0.608	17124552	INTS4P2	−0.531	−0.309
16765809	MMP19	0.624	0.348	17014208	SYTL3	−0.513	−0.478
17047411	POR	0.617	0.344	17011895	NT5DC1	−0.500	−0.347
16843187	UTP6	0.599	0.322	16774789	SETDB2	−0.491	−0.374
16719882	FUOM	0.596	0.414	16761212	CLEC2B	−0.483	−0.408
16883624	IL1R2	0.591	0.307	17007795	DEF6	−0.478	−0.533
16915044	RTF2	0.591	0.367	16761518	TAS2R31	−0.464	−0.655
16746696	ADIPOR2	0.547	0.349	16709108	MXI1	−0.462	−0.366
16853399	COLEC12	0.544	0.912	16838746	LOC100129503	−0.413	−0.442
16672946	FCRLA	0.544	0.386	16739979	PYGM	−0.392	−0.325
17076063	GSR	0.534	0.390	16687598	PARS2	−0.357	−0.300
16763246	GXYLT1	0.505	0.316	16777756	SLC46A3	−0.346	−0.373
16923666	KRTAP10-11	0.504	0.306	17114520	ARHGEF6	−0.340	−0.652
16817811	PPP4C	0.497	0.312				
16954827	NT5DC2	0.472	0.486				
16829885	PSMB6	0.469	0.350				
16993506	CNOT6	0.425	0.344				
17107907	NSDHL	0.419	0.314				
16829738	CTNS	0.419	0.329				
16734644	OR7E12P	0.417	0.427				
17013784	ARMT1	0.396	0.305				
16818114	HSD3B7	0.383	0.340				

Table S3. Pathways (35) associated with DEGs in monocytes of patients with ESRD. These pathways are listed based on FWER *p*-value ($p < 0.05$, FDR < 0.25).

Enriched Pathways*	NOM *p*-value	NES	FDR *q*-value	**FWER *p*-value
APICAL JUNCTION	<0.0001	1.647921	0.16900006	0.05
GLYCOLYSIS	<0.0001	1.594081	0.106500015	0.094
OXIDATIVE PHOSPHORYLATION	<0.0001	1.556408	0.085666634	0.094
CHOLESTEROL HOMEOSTASIS	<0.0001	1.539448	0.07524997	0.094
EPITHELIAL MESENCHYMAL TRANSITION	<0.0001	1.537626	0.069	0.094
MTORC1 SIGNALING	<0.0001	1.525755	0.07433329	0.151
INTERFERON ALPHA RESPONSE	<0.0001	1.493075	0.069999985	0.151
PROTEIN SECRETION	<0.0001	1.483183	0.06674993	0.151
KRAS SIGNALING UP	<0.0001	1.478995	0.07811108	0.151
FATTY ACID METABOLISM	<0.0001	1.476353	0.074699946	0.151
ANDROGEN RESPONSE	<0.0001	1.470947	0.071909	0.151
ADIPOGENESIS	<0.0001	1.470279	0.06958327	0.151
XENOBIOTIC METABOLISM	<0.0001	1.457141	0.06761535	0.151
ESTROGEN RESPONSE LATE	<0.0001	1.455282	0.06592856	0.151
REACTIVE OXYGEN SPECIES PATHWAY	<0.0001	1.45284	0.064466625	0.151
UV RESPONSE UP	<0.0001	1.448635	0.063187435	0.151
IL6 JAK STAT3 SIGNALING	<0.0001	1.444561	0.06205876	0.151
NOTCH SIGNALING	<0.0001	1.44141	0.06947479	0.237
PEROXISOME	<0.0001	1.431104	0.06813402	0.237
HYPOXIA	<0.0001	1.423977	0.06692729	0.237
MYOGENESIS	<0.0001	1.418742	0.06583552	0.237
BILE ACID METABOLISM	<0.0001	1.418112	0.06484298	0.237
INTERFERON GAMMA RESPONSE	<0.0001	1.405151	0.063936815	0.237
P53 PATHWAY	<0.0001	1.404928	0.0686269	0.29
HEDGEHOG SIGNALING	<0.0001	1.40372	0.06764185	0.29
INFLAMMATORY RESPONSE	<0.0001	1.382336	0.07411718	0.338
ANGIOGENESIS	<0.0001	1.373581	0.07300168	0.338
ALLOGRAFT REJECTION	<0.0001	1.352267	0.07196596	0.338
APOPTOSIS	<0.0001	1.34994	0.07100157	0.338
MITOTIC SPINDLE	<0.0001	1.344484	0.07576416	0.338
HEME METABOLISM	<0.0001	1.344062	0.08093316	0.338
COAGULATION	<0.0001	1.342428	0.07977895	0.338
COMPLEMENT	<0.0001	1.33105	0.084512904	0.338
TNFA SIGNALING VIA NFKB	<0.0001	1.330804	0.0833213	0.338
IL2 STAT5 SIGNALING	<0.0001	1.328439	0.0984471	0.338

* The GSEA outputs were filtered based on a normalized enrichment score (NES) > 1.3.
**FWDR (Familywise Error Rate) is the probability of a coming to at least one false conclusion in a series of hypothesis tests.

Table S4. The enriched pathways and their associated genes in commonly upregulated DEGs in IS-treated monocytes and ESRD patient monocytes identified via analysis of a DAVID functional annotation and KEGG mapper. This list was ordered based on a log2 fold value of ESRD patients (*p* < 0.05, FDR < 0.25).

Pathway	Gene Name (Symbol)	ESRD (log2FC)	IS (log2FC)
**Metabolic pathway***	FBP1	3.452914476	0.454519152
	PGD	2.687466734	0.71364921
	CYP27A1	4.076360023	1.270835575
	NDST1	2.380248485	0.308801695
	G6PD	1.816466603	0.4641929
	MGST1	2.166759325	0.854075059
	ACER3	1.907401067	0.417765426

	TALDO1	1.693009515	0.378480983
	GBGT1	1.513536184	0.308002446
	GPAT3	1.71972151	0.352222576
	CA2	1.293695168	0.377834633
	GALNT1	1.08741439	0.342558624
	GCNT1	1.018434463	0.404784583
	SQOR	1.206086396	0.34542446
	ME1	0.958219089	0.801311426
	GSR	0.534106451	0.389596344
	XYLT1	0.676391365	0.514482369
	NSDHL	0.418873969	0.313619089
	HSD3B7	0.383121999	0.339774652
**Phagosome**	CD14	6.173316039	0.309943993
	C3	2.147539172	0.507833879
	CD209	1.479383065	0.43460869
	ITGAV	0.841959543	0.471796382
	CTSL	1.346141514	0.468885076
	MRC1	0.743094687	1.184179084
	NCF1	0.956874752	0.801864253
**Cytokine-cytokine receptor interaction**	CCR1	4.371532236	0.302223697
	CXCL8	6.59448077	0.317102853
	ACVR1B	0.961494703	0.316625232
	IL1R2	0.590989599	0.306877414
	IL3RA	1.202899962	0.763965849
	TNF	1.835281535	0.696791124
**NF-κB signaling pathway**	CD14	6.173316039	0.309943993
	CXCL8	6.59448077	0.317102853
	TRIM25	1.590075259	0.347105934
	TNF	1.835281535	0.696791124
**TNF signaling pathway**	MMP14	1.128527435	0.648830575
	TNF	1.747786532	0.396235072
**Metabolism of xenobiotics by cytochrome P450**	CYP1B1	3.205113913	1.573801494
	MGST1	2.166759325	0.854075059

*Metabolic pathways also included Carbon metabolism, Pentose phosphate pathway, Superoxide-generating NADPH oxidase activity, NADPH metabolic process and Oxidation-reduction process.

Table S5. Primers for validated genes.

Gene Name	Primer Sequence (5′–3′)
**Human CYP27A1**	Forward: CAAGGCTGATCCAGAAGTACAA
	Reverse: TGCAGGCCCACTTTCTTATT
**Human ME1**	Forward: CCCATCCTTCAGCTACCATAC
	Reverse: CTCTCCTTTCAGACCTTCTCAC
**Human PGD**	Forward: TGGCCCTTTGCCCTATTT
	Reverse: GGCTCTCACAGAGCTACTTTAC
**Human XYLT1**	Forward: GTCTCTCTCTCTCTCGGTTTCT
	Reverse: GCTTTCTTTGCGTGGTGTTC
**Human G6PD**	Forward: ACCTCATGGTGCTGAGATTTG
	Reverse: GCTCCTTGAAGGTGAGGATAAC
**Human TALDO1**	Forward: CGCAAGGCTCTCCTTTGATA
	Reverse: ATTCCTTCCCAGGTTGATGAC

**Human MGST1**	Forward: CCCACCTGAATGACCTTGAA<br>Reverse: ATCCGTGCTCCGACAAATAG
**Human MMP14**	Forward: GCCCAATGGAAAGACCTACTT<br>Reverse: CCCTTCCCAGACTTTGATGTT
**Human CYP1B1**	Forward: TGCCTGTCACTATTCCTCATGCCA<br>Reverse: ATCAAAGTTCTCCGGGTTAGGCCA
**Human TNF-α**	Forward: AGCCCATGTTGTAGCAAACC<br>Reverse: TGAGGTACAGGCCCTCTGAT
**Human Actin**	Forward: TGCCTGTCACTATTCCTCATGCCA<br>Reverse: ATCAAAGTTCTCCGGGTTAGGCCA
